# Does Variation of the Inter-Domain Linker Sequence Modulate the Metal Binding Behaviour of *Helix pomatia* Cd-Metallothionein?

**DOI:** 10.3390/ijms17010006

**Published:** 2015-12-22

**Authors:** Selene Gil-Moreno, Elena Jiménez-Martí, Òscar Palacios, Oliver Zerbe, Reinhard Dallinger, Mercè Capdevila, Sílvia Atrian

**Affiliations:** 1 Departament de Química, Facultat de Ciències, Universitat Autònoma de Barcelona, E-08193 Cerdanyola del Vallès, Spain; selenebdn89@gmail.com (S.G.-M.); oscar.palacios@uab.cat (O.P.); merce.capdevila@uab.cat (M.C.); 2 Departament de Genètica, Facultat de Biologia, Universitat de Barcelona, Av. Diagonal 643, E-08028 Barcelona, Spain; ejimenezmarti@gmail.com; 3 Institute of Organic Chemistry, University of Zurich, 8057 Zurich, Switzerland; zerbe@oci.uzh.ch; 4 Institute of Zoology, University of Innsbruck, Technikerstraße 25, A-6020 Innsbruck, Austria; reinhard.dallinger@uibk.ac.at

**Keywords:** Cd-isoform, domain linker sequence, *Helix pomatia*, metallothionein, metal binding

## Abstract

Snail metallothioneins (MTs) constitute an ideal model to study structure/function relationships in these metal-binding polypeptides. *Helix pomatia* harbours three MT isoforms: the highly specific CdMT and CuMT, and an unspecific Cd/CuMT, which represent paralogous proteins with extremely different metal binding preferences while sharing high sequence similarity. Preceding work allowed assessing that, although, the Cys residues are responsible for metal ion coordination, metal specificity or preference is achieved by diversification of the amino acids interspersed between them. The metal-specific MT polypeptides fold into unique, energetically-optimized complexes of defined metal content, when binding their cognate metal ions, while they produce a mixture of complexes, none of them representing a clear energy minimum, with non-cognate metal ions. Another critical, and so far mostly unexplored, region is the stretch linking the individual MT domains, each of which represents an independent metal cluster. In this work, we have designed and analyzed two HpCdMT constructs with substituted linker segments, and determined their coordination behavior when exposed to both cognate and non-cognate metal ions. Results unequivocally show that neither length nor composition of the inter-domain linker alter the features of the Zn(II)- and Cd(II)-complexes, but surprisingly that they influence their ability to bind Cu(I), the non-cognate metal ion.

## 1. Introduction

Metallothioneins (MTs) are a super-family of mostly small, ubiquitous, but highly heterogeneous, proteins that coordinate heavy-metal ions owing to the metal-thiolate bonds contributed by their abundant cysteine residues (recent reviews in [1,2]). They have been traditionally associated with different biological roles mainly related to physiological metal (Zn and Cu) homeostasis and/or to toxic heavy metal chelation, but also to different stress responses, such as free radical scavenging. It has been hypothesized that this multitude of possible functions may be the basis of the high heterogeneity of the MT proteins reported up to now, so that MTs may have evolved differently in certain groups of organisms according to precise physiological requirements. Hence, the extraordinary diversity of MT isoforms -MTs are polymorphic in almost all organisms analyzed up to now—along all kinds of taxa seem to be related to their plasticity to perform a great multiplicity of functions [3]. At first, several classifications of this heterogeneous group of proteins had been first proposed on the basis of sequence similarity and taxonomic criteria [4,5], but most significantly, our group later proposed a functional classification of MTs founded on the analysis of their preference for divalent metal ion coordination, grouping them into the so-called Zn-thioneins (accounting for both Zn(II) or Cd(II)-binding MTs), and the so called Cu-thioneins (accounting for monovalent ion binding MTs). Each type of MT is characterized by yielding unique, well-folded, homometallic complexes when it coordinates its cognate metal ion [6]. Although originally this classification only proposed these two MT categories [7], it was later extended to a step-wise gradation between extreme, or genuine, Zn- (or divalent metal-ions)-thioneins and Cu-thioneins [8].

*Gastropoda* pulmonates is one of the Mollusca classes with a higher number of species, and they constitute an ideal model system to study the structure/function relationship and the evolutionary differentiation of polymorphic MTs. The different MT isoforms combine two valuable properties that allow to precisely recognize the features that confer the Zn- or Cu-thionein character to an MT polypeptide: the paralogous proteins are highly specialized for binding distinct metal ions while retaining high sequence similarities. Hence, the best characterized snail MT systems, those of the terrestrial snails *Helix pomatia* and *Cantareus aspersus* include three paralogous MT peptides with different metal binding preferences: the CdMT and CuMT isoforms which, respectively, bind cadmium and copper with high specificity, and an unspecific Cd/CuMT isoform that was isolated as a mixed Cd and Cu native complex. The CdMT and CuMT proteins were first extensively characterized in the species *Helix pomatia* [9], whereas the unspecific Cd/CuMT was initially isolated from cadmium-intoxicated garden snails (*Cantareus aspersus*) [10]. Nevertheless, its presence was later also corroborated in *H. pomatia* [11]. Since the synthesis of the *H. pomatia* Cd-specific isoform (HpCdMT) was shown to be inducible by cadmium food supplementation, and since it yielded homonuclear Cd_6_-complexes, a metal detoxification role in the snail digestive tract was assigned to this peptide [12,13]. Contrarily, the Cu-specific isoform (HpCuMT), natively isolated as homonuclear Cu_12_-complexes, is constitutively synthesized in the rhogocytes, which suggested a possible involvement in hemocyanin synthesis through storage and delivery of the required copper [14]. Further data from recombinantly-synthesized metal-complexes allowed to demonstrate that variations of the amino acid sequence interspersed between their fully conserved cysteines had led to the metal binding specificity these two *H. pomatia* MTs [11]. More recently, studies of the metal-binding behavior towards either cognate or non-cognate metal ions revealed that MT biosynthesis in the presence of the former renders unique, energetically optimized complexes, which is what outlines their metal specificity or preference. In contrast, the binding of non-cognate metal ions results in a mixture of complexes, with varied stoichiometries and folds [15]. Thus, the thermodynamic stability of the metal-MT complexes appears not exclusively related to their metal-thiolate bonds, as could have been theorized from strict chemical considerations, but is rather determined by the nature of the non-coordinating residues of each MT sequence [15].

In addition to Cys patterns and the nature of the intercalated residues, a third element that may modulate the binding behavior of an MT polypeptides are the linker stretches between the domains (metal-clusters). Their length and composition may influence the stability and independency of the metal-MT structural domains. This is a rather unexplored aspect of MT structures, mainly because the number of 3D structures available is still limited and, therefore, does not provide a sound statistical basis to study this aspect. In fact, among the 16 MT structures available in PDB [2], only one—the rat liver Zn_2_, Cd_5_–MT2 complex—reveals the relative orientation of the two domains. This structure displays the paradigmatic dumbbell shape that vertebrate MTs yield when they coordinate divalent metal ions: the N-terminal segment (β domain), with 9 cysteines in Cys–X–Cys arrays, which binds three M(II) ions, and the C-terminal segment (α domain), with 11 cysteines, which binds four M(II) ions [16]. In all the other proteins, the putative independent domains have been solved independently by NMR, and were assumed to be connected more or less flexibly by the linker residues. However, for some MTs despite the absence of resolved 3D structures, some clear data have been reported, about the influence of both the linker composition and the N-term and C-term MT flanking regions for their metal binding capabilities, such as in the case of arsenic chelation by *Fucus vesiculosus* MT [17], cadmium scavenging by the type 2 *Quercus suber* QsMT [18] or, most recently, copper coordination by the two fungal *Cryptococcus neoformans* CnMT1 and CnMT2 isoforms [19].

In this report, we aimed at investigating the influence of the amino acid sequence in the linker connecting the two nine-Cys moieties of HpCdMT for the stoichiometry and folding of the corresponding Zn(II), Cd(II), or Cu(I) complexes. Although no crystal or solution structure of the Cd-HpCdMT complex is yet available (work is in progress), the results from its spectroscopic characterization fully support the existence of two domains constituting separate Cd_3_Cys_9_ clusters [20], as observed for the marine crustaceans [21,22] and the *C. elegans* MTs [23]. To this end, two mutant HpCdMT proteins with prolonged linkers (eight instead of the two native residues) were designed and expressed in *E. coli*. Thereafter, the metal binding behavior of these two mutants (called HpCdMcMT and HpCdPlMT from now on) was assessed for recognizing both their cognate metal ions (*i.e.*, Zn(II) and Cd(II)), but also the non-cognate monovalent Cu(I) ions, and all the data were compared with those from the wild-type HpCdMT isoform.

## 2. Results and Discussion

### 2.1. The HpCdMcMT and HpCdPlMT Recombinant Peptides

**Figure 1:**
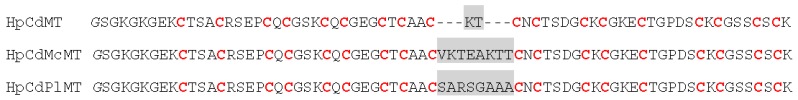
Sequence alignment of the recombinant proteins studied in this work: the constructs HpCdMcMT and HpCdPlMT are aligned with the HpCdMT wild-type form. The Cys residues are written in red, and the linker residues are shaded in grey. The initial Gly, which is a remainder from the thrombin cleavage site, is printed in italics.

**Figure 2:**
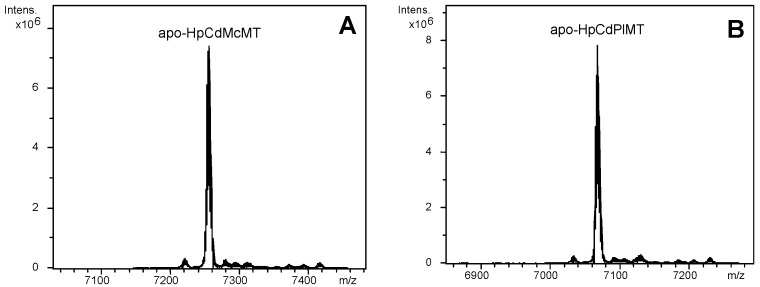
Deconvoluted ESI-MS spectra of the recombinant preparations of (**A**) HpCdMcMT; and (**B**) HpCdPlMT purified from bacterial cultures grown under Zn-supplementation, analyzed at acid pH (2.4).

The two HpCdMT mutants designed contained longer linkers than the -KT- dipeptide of the wild type protein: one of them—that of HpCdMcMT—reproduces the one of another gastropod MT, *M. crenulata*, and exhibits a clear polar character (-VKTEAKTT-) [24]. The other linker—that of HpCdPlMT—derives from a plant MT (the wheat Ec-1 protein), and is of clear apolar composition (-SARSGAAA-) [25]. DNA sequencing of the HpCdMcMT- and HpCdPlMT-coding pGEX-4T-1 constructs ruled out any nucleotide mutation, and confirmed that the cDNAs were cloned in correct frame. After expression in *E. coli* and purification, acidification of the recombinant Zn-HpCdMcMT and Zn-HpCdPlMT samples yielded the corresponding apo-forms, with respective molecular masses of 7254.7 and 7066.6 Da, in accordance with the respective theoretical values of 7255.7 and 7067.9 Da (Figure 1 and Figure 2). This confirmed the correctness of both synthesized proteins.

### 2.2. Zn and Cd Binding Abilities of HpCdMcMT and HpCdPlMT

**Figure 3:**
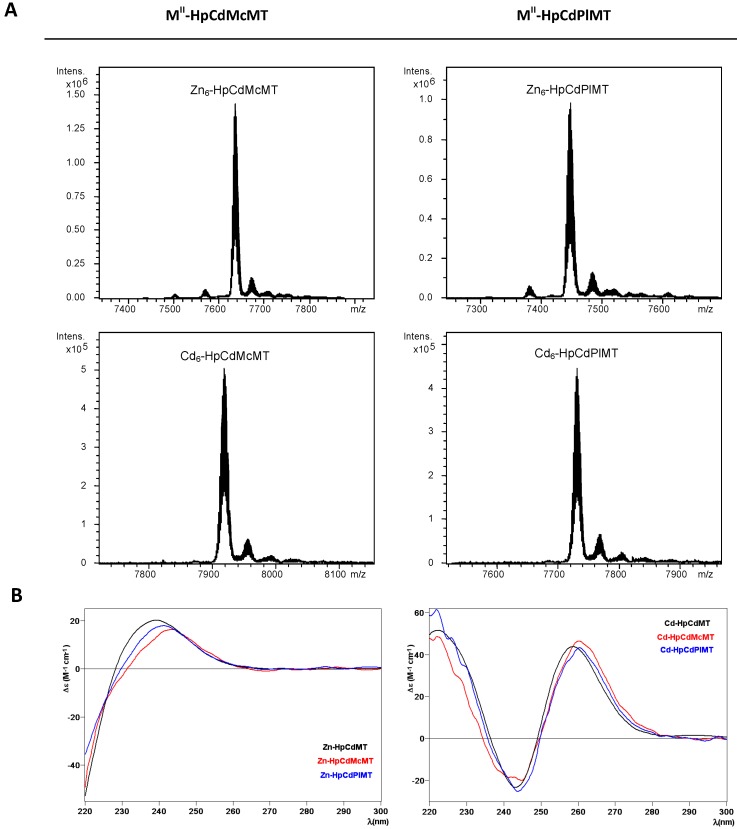
Analysis of the Zn- and Cd-HpCdMcMT and -HpCdPlMT complexes. (**A**) Deconvoluted ESI-MS spectra of the recombinant preparations of HpCdMcMT and HpCdPlMT purified from Zn- and Cd-supplemented cultures, analyzed at neutral pH (7.0); (**B**) CD spectra of the corresponding recombinant preparations. For comparative purposes, the CD spectra of the recombinant preparations yielded by the wild-type HpCdMT protein [11] have been included.

Both HpCdMcMT and HpCdPlMT polypeptides synthesized in Zn-supplemented *Escherichia coli* cultures folded into unique Zn_6_-complexes, (*cf.* the ESI-MS spectra shown in Figure 3A, where only very minor, negligible accompanying peaks are detected, attributable to frequently observed NH_4_^+^ adducts, and Table 1). The syntheses in Cd-supplemented cultures equally yielded almost unique peaks corresponding to the Cd_6_-complexes of both peptides, as identified in the respective ESI-MS analyses at neutral pH (Figure 3A and Table 1). These results fully coincide with the Zn- and Cd-species afforded by the wild-type HpCdMT synthesized under equivalent conditions, that affords unique M(II)_6_ complexes, as we demonstrated in [11]. Analysis of the CD spectra of the Zn- and Cd-preparations of HpCdMcMT and HpCdPlMT totally confirmed that these two mutants exhibit equivalent folds when coordinating Zn(II) ions, and also when coordinating Cd(II) ions, which are also practically indistinguishable from those of the respective wild-type HpCdMT complexes (Figure 3B). For example, the Zn-MT complexes show the typical Gaussian band centred at *ca*. 240 nm, while the Cd-MT species display the exciton coupling envelop at *ca*. 250 nm characteristic of the Zn- and Cd-thiolate chromophores.

**Table 1:** Analytical characterization of the recombinant Zn(II)- and Cd(II)-complexes of the HpCdMT mutants studied in this work. For comparative purposes, data for the recombinantly-synthesized wild-type HpCdMT are included [11].

MT	ICP-AES ^a^	Neutral ESI-MS ^b^	Experimental MM ^c^	Calculated MM ^d^
HpCdMT [11]	5.8	Zn_6_-MT	7005	7005.6
HpCdMcMT	5.9	Zn_6_-MT	7635	7635.6
HpCdPlMT	6.0	Zn_6_-MT	7448	7448.3
HpCdMT [11]	6.2	Cd_6_-MT	7287	7287.8
HpCdMcMT	6.1	Cd_6_-MT	7917	7917.7
HpCdPlMT	6.6	Cd_6_-MT	7730	7730.4

^a^ Zn(II)-to-peptide ratio calculated from S and Zn content (ICP-AES data); ^b^ The deduced Zn(II)-species were calculated from the mass difference between the holo- and apo-peptides; ^c^ experimental molecular masses corresponding to the detected M(II)-complexes. The corresponding ESI-MS spectra are shown in Figure 3; ^d^ theoretical molecular masses corresponding to the M(II)-complexes.

**Figure 4:**
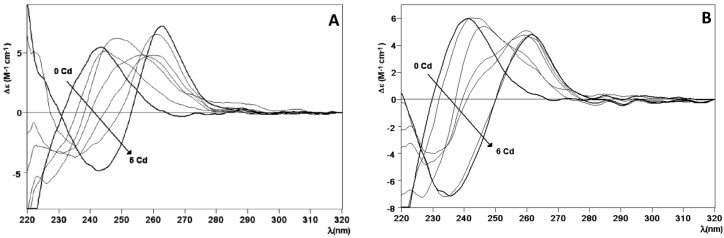
Zn(II)/Cd(II) replacement reaction of the Zn-HpCdMcMT and Zn-HpCdPlMT complexes. (**A**) CD spectra of a 10 µM solution of the Zn-HpCdMcMT sample titrated with CdCl_2_ at neutral pH up to six Cd(II) equivalent; (**B**) CD spectra of a 10 µM solution of the Zn-HpCdPlMT sample titrated with CdCl_2_ at neutral pH, up to six Cd(II) equivalent.

Furthermore, the Zn^2+^/Cd^2+^ displacement process in Zn_6_-HpCdMcMT and Zn_6_-HpCdPlMT was a straight reaction that exclusively yielded Cd_6_-complexes after the addition of 6 Cd^2+^ eq (Figure 4A,B, respectively), in agreement with the behavior in wild-type Zn_6_-HpCdMT [15]. Most significantly, not only the final step of this reaction was the same, and also equivalent to the respective recombinant Cd-complexes, but CD spectra recorded at progressive stages of the reaction revealed identical profiles (*cf*. Figure 4). These basically consist in the evolution of the initial Gaussian band at *ca*. 240 nm characteristic of the Zn-complexes to the exciton-coupling signal centered at *ca*. 250 nm, typical of Cd-complexes. This suggests that the Zn(II)/Cd(II) substitution proceeds in an almost parallel way in all three cases, *i.e.*, for the wild-type HpCdMT and for the two mutant forms.

Therefore, it can be concluded that the different composition of the amino acid sequence linking the two putative domains of the HpCdMT proteins does affect neither the stoichiometry nor the folding of the complexes formed when coordinating divalent metal ions. To conclude, the linker length and compositions do not seem to alter the binding behavior towards a cognate metal ion.

### 2.3. Cu Binding Abilities of HpCdMcMT and HpCdPlMT

Before going into the details of the Cu(I) binding analysis of the two mutant constructs HpCdMcMT and HpCdPlMT, it is worth remembering that the data of the previous Cu(I) binding study performed with the wild-type HpCdMT form already exhibited a high degree of complexity, typical of the recombinant samples obtained when synthesizing a MT protein (here HpCdMT) in the presence of its non-cognate metal ion (here Cu(I)) [15]. As previously described [26], we perform two types of Cu-supplemented productions: at standard and at low aeration conditions. This responds to the known influence of culture oxygenation on the amount of internal copper in bacteria, which determines the composition of the final Cu-species. But unfortunately, several efforts to purify HpCdMcMT and HpCdPlMT from *E. coli* cultures grown at low oxygen conditions were not successful. Contrarily, the synthesis of both polypeptides performed at regular oxygen conditions yielded preparations that allowed their analysis by ESI-MS and CD, and facilitated the comparison of all their features with those of the complexes obtained from HpCdMT.

**Figure 5:**
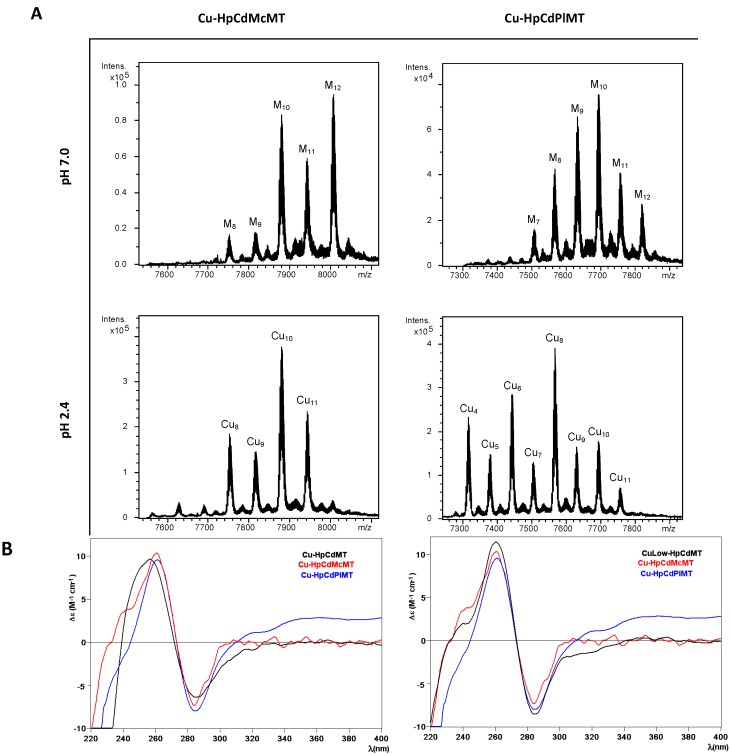
Analysis of the Cu-HpCdMcMT and Cu-HpCdPlMT complexes. (**A**) Deconvoluted ESI-MS spectra of the recombinant preparations of HpCdMcMT and HpCdPlMT purified from Cu-supplemented cultures, analyzed at neutral pH (7.0); (**B**) CD spectra of the same recombinant preparations. For comparative purposes, the CD spectra of the corresponding recombinant preparations yielded by the wild-type HpCdMT protein [15] have been included.

The first noticeable observation was that the composition of the Cu-HpCdMcMT and Cu-HpCdPlMT purified samples was significantly different, and in turn different from that of the wild-type Cu-HpCdMT preparations [15]. For example, the ESI-MS spectra of the Cu-HpCdMcMT sample recorded at neutral pH exhibited two clear major peaks corresponding to M_12_- and M_10_-HpCdMcMT complexes, an intermediate M_11_-HpCdMT and very minor M_9_- and M_8_-HpCdMcMT species (Figure 5A, Table 2), where M can be either Zn(II) or Cu(I) because the similarity of their atomic masses. When this same preparation was analyzed at pH 2.4—which allows the release of all bound Zn(II) but not of Cu(I) [27,28]—one major peak corresponding to homonuclear Cu_10_-HpCdMT together with minor peaks of Cu_11_-, Cu_8_- and Cu_9_-complexes were observed (Figure 5A, Table 2). Taking into consideration that the ICP-AES analyses of the purified Cu-HpCdMcMT sample yielded an average metal content of 1.32 Zn and 12.25 Cu per MT, the most straightforward explanation for these results is to assume the presence of a mixture of major homonuclear complexes (Cu_11_-, Cu_10_-, Cu_9_- and Cu_8_-HpCdMcMT) together with some minor amounts of heteronuclear CuZn-HpCdMcMT species, mainly accounting for the M_12_-HpCdMcMT species. On the other hand, a slightly poorer Cu-binding capacity can be attributed to HpCdPlMT for the following reasons: First, the ICP-AES analysis indicated a Zn:Cu mean content in the purified preparation of 2.17 Zn:7.87 Cu, corresponding to higher Zn:Cu ratio than in the HpCdMcMT sample. Second, the Cu-HpCdPlMT sample presents a more complex mixture, in terms of the number of ESI-MS detected species, both at neutral and acid pH. Thus, the ESI-MS spectrum at pH 7.0 allows the detection of a major M_10_-HpCdPlMT peak, followed by M_9_-, M_11_-, M_8_-, M_12_-, and M_7_-HpCdPlMT species, in decreasing order of intensity. This sample exhibited a mixture of major Cu_8_-, and then minor Cu_6_-, Cu_4_-, and all the rest of peaks between Cu_4_- and Cu_11_-HpCdPlMT when analyzed at acidic ESI-MS, which would point to a continuum of heterometallic ZnCu-HpCdPlMT species (Figure 5A, Table 2).

**Table 2:** Analytical characterization of the recombinant complexes obtained from Cu supplemented cultures of the HpCdMT mutants studied in this work. All data for the two mutant proteins correspond to normal aerated cultures, since no complexes could be recovered from low aeration conditions. For comparative purposes, data for the wild-type HpCdMT, recombinantly-synthesized in Cu-supplemented cultures grown at both aeration conditions, are included [15].

MT	ICP-AES ^a^	Neutral ESI-MS ^b^	MM_Exp_^c^	MM_Theor_^d^	Acidic ESI-MS ^b^	MM_Exp_^c^	MM_Theor_^d^
HpCdMT Normal aeration [15]	2.6 Zn 1.9 Cu	**M_5_-MT**	6932	6938.1			
		M_4_-MT	6867	6875.5	**apo-MT**	6623	6625.5
		M_6_-MT	6996	7000.6	Cu_4_-MT	6873	6875.5
		M_7_-MT	7062	7063.2	Cu_5_-MT	6930	6938.1
		M_8_-MT	7124	7125.7			
HpCdMT Low aeration [15]	0.8 Zn 8.3 Cu	**M_10_-MT**	7251	7250.8	**Cu_8_-MT**	7120	7,125.7
		M_11_-MT	7313	7313.4	Cu_10_-MT	7248	7,250.8
		M_12_-MT	7379	7375.9	Cu_11_-MT	7312	7313.4
		M_8_-MT	7122	7125.9	Cu_9_-MT	7184	7188.3
		M_9_-MT	7182	7188.3	Cu_5_-MT	6929	6938.1
HpCdMcMT	1.3 Zn 12.3 Cu	**M_12_-MT**	8006	8005.8	**Cu_10_-MT** Cu_11_-MT Cu_8_-MT Cu_9_-MT	7879 7942 7753 7816	7880.7 7943.3 7755.6 7818.2
		**M_10_-MT**	7878	7880.7			
		M_11_-MT	7942	7943.3			
		M_9_-MT	7816	7818.2			
		M_8_-MT	7753	7755.6			
HpCdPlMT					**Cu_8_-MT**	7566	7568.3
		**M_10_-MT**	7692	7693.4	Cu_6_-MT	7442	7443.2
		M_9_-MT	7631	7630.9	Cu_4_-MT	7316	7318.1
	2.2 Zn	M_8_-MT	7566	7568.3	Cu_10_-MT	7692	7693.4
	7.9 Cu	M_11_-MT	7755	7755.9	Cu_9_-MT	7629	7630.9
		M_12_-MT	7818	7818.5	Cu_5_-MT	7377	7380.7
		M_7_-MT	7505	7505.8	Cu_7_-MT	7503	7505.8
					Cu_11_-MT	7755	7755.9

^a^ Zn(II) and Cu(I)-to-peptide ratio calculated from S, Zn, and Cu content (ICP-AES data); ^b^ the deduced species (M = Zn or Cu) were calculated from the mass difference between the holo- and the respective apo-peptides. The major species are indicated in bold, and the rest are in decreasing order of ESI-MS peak intensity; ^c^ experimental molecular masses corresponding to the detected complexes. The corresponding ESI-MS spectra are shown in Figure 5; ^d^ theoretical molecular masses corresponding to the metal-complexes.

Finally, comparison of these results with those from the wild-type HpCdMT protein also differed considerably (included in Table 2 for comparative purposes). Synthesis of Cu-HpCdMT yielded very poor results when performed in normally aerated cultures [15], only allowing recognizing major M_5_-HpCdMT and minor M_4_- to M_8_-complexes that contained Cu_4_- or Cu_5_-cores. In fact, the results for the two mutant constructs analyzed in this work resembled far more those of Cu-HpCdMT produced in low aerated cultures, this means in the presence of higher intracellular copper levels, because in that case also major M_10_-HpCdMT, together with minor M_8_- to M_12_-complexes were identified by neutral ESI-MS, which were composed of major Cu_8_-cores [15]. However, despite the diversity of results about the species composition yielded from the three HpCdMT proteins when coordinating Cu(I), they all gave rise to comparable CD signals, both in shape and intensity, which display the typical envelope of the Cu-MT complexes with absorption maxima at *ca*. 260 nm and minima at *ca*. 280 nm. The CD fingerprint of the Cu-HpCdMcMT and Cu-HpCdPlMT samples was more similar to that of the Cu-HpCdMT synthesized at low aeration [15] (Figure 5B), in concordance with the above-commented species composition.

Therefore, it can be unambiguously concluded that the inter-domain linker features (length and composition) greatly influences the Cu(I) binding behavior of the HpCdMT protein, so that the capacity to bind Cu(I) for the mutant HpCdMcMT and HpCdPlMT constructs, with eight amino acid-long linkers between the ninth and tenth Cys in their sequence, appears considerably enhanced in comparison with HpCdMT whose linker contains only two residues between the homologous Cys residues (*cf.* Figure 1). However, the differences in Cu-binding capabilities between HpCdMcMT and HpCdPlMT reveals that not only the length, but also the composition of the linker is important. Hence, the linker of the HpCdMcMT construct, containing two lysine, one glutamic acid and three threonine residues, seems to favor the formation of higher nuclearity homonuclear Cu(I) complexes, over the highly apolar HpCdPlMT linker, that is composed of only one arginine and two serine residues and that also contains five apolar amino acids (four alanine and a glycine).

## 3. Experimental Section

### 3.1. Construction and Cloning of the cDNAs Encoding the HpCdMT Linker Mutants

Two HpCdMT mutants were designed to replace the only two residues (-KT-) that act as a linker between the two nine-Cys domains in the HpCdMT sequence with the corresponding linkers from the (giant keyhole limpet) *M. crenulata* MT [24] (-VKTEAKTT-; HpCdMcMT protein) or from wheat (*T. aestivum*) Ec-1 MT [25] (-SARSGAAA-; HpCdPlMT protein) (Figure 1). The cDNAs encoding for these sequences were designed on the basis of the flanking regions in wild-type HpCdMT cDNA, and to encode the new linkers in the corresponding limpet or wheat cDNAs. Additionally, the restriction sites for *BamH*I and *Xho*I were added to the 5’ and 3’ ends respectively for cloning purposes (Supplementary Materials, Figure S1). The HpCdMcMT and HpCdPlMT coding sequences designed this way were purchased as synthetic DNAs from IDT & Conda Labs (Spain). After PCR amplification (35 cycles: 95 °C 30 s, 50 °C 30 s, and 72 °C 30 s, using Expand High Fidelity (Roche) thermostable DNA polymerase) of the synthetic cDNAs using the flanking primers: 5’-TTTATTGGATCCGGTAAGGG-3’ (HpCdMcMT forward); 5’-TTTCTCGAGTTACTTACAGG-3’ (HpCdMcMT reverse); 5’-TTTATTGGATCCGGCAAAGGG-3’ (HpCdPlMT forward); and 5’-TTTCTCGAGTTATTTGCAAG-3’ (HpCdPlMT reverse). They were subsequently digested with *BamH*I and *Xho*I restriction enzymes, and the resulting products were ligated in-frame (DNA ligation kit, Takara Bio, Kusatsu, Shiga, Japan) in the pGEX-4T-1 (Amersham-GE Healthcare Europe, Cerdanyola del Valles, Spain) *E. coli* expression vector, which yields GST-fusion proteins. DNA sequencing allow confirming all the DNA constructs (ABIPRISM 310, Applied Biosystems, Foster City, CA, USA), using BigDye Terminator. The *E. coli* MachI strain was used for cloning and sequencing. The expression plasmids were then transformed into the protease-deficient strain BL21 for protein synthesis. The construction of the pGEX plasmid encoding for the wild type HpCdMT isoform has been previously described [11].

### 3.2. Synthesis and Purification of the Recombinant Zn-, Cd-, and Cu-Complexes of the HpCdMT Linker Mutants

All the purifications of the metal-MT complexes were carried out as reported in [29] for the wild-type HpCdMT isoform, which ensured full comparative results. Hence, the GST-HpCdMT fusions were produced in 5-L cultures (Luria Bertani medium) of transformed *E. coli* BL21 bacteria. Induction of gene expression was achieved with 100 µM (final concentration) of isopropyl β-d-thiogalactopyranoside (IPTG). After 30 min of induction, 300 µM ZnCl_2_ or 500 µM CuSO_4_ (final concentration) were supplemented to the cultures, which grew for a further 2.5 h, for the synthesis of the respective metal complexes. The Cu-cultures were grown both under normal (1-L medium in a 2-L Erlenmeyer flask, at 250 rpm) and low oxygen conditions (1.5-L medium in a 2-L Erlenmeyer flask at 150 rpm). It is well known that the culture aeration determines the amount of intracellular copper in the host cells available for the newly synthesized MTs [26].

Harvesting and centrifugation of the grown cells renders a cell mass that, resuspended in ice-cold PBS (1.4 M NaCl, 27 mM KCl, 101 mM Na_2_HPO_4_, 18 mM KH_2_PO_4_) with 0.5% *v*/*v* β-mercaptoethanol, is disrupted by sonication (20 s pulses for 5 min). All solutions used were oxygen-purged by saturating them with pure-grade argon to prevent metal-MT oxidation. The suspension was centrifuged at 12,000× *g* for 30 min, and the incubation of the resulting supernatant (gentle agitation for 60 min at room temperature) with Glutathione-Sepharone 4B (GE Healthcare) allowed batch affinity purification of the GST-HpCdMT species. The MT portion was recovered after thrombin cleavage (10 µ per mg of fusion protein at 17 °C over-night). The solution containing the cleaved metal-MT complexes was concentrated by Centriprep Microcon 3 (Amicon, cut-off of 3 kDa, Merck-Millipore, Darmstadt, Germany) centrifugation. The final metal complexes were purified through FPLC size-exclusion chromatography in a Superdex75 column (GE Healthcare) equilibrated with 50 mM Tris-HCl, pH 7.0 and run at 0.8 mL·min^−1^. Absorbances at 254 and 280 nm signaled the fractions to be collected and analyzed for protein content.

### 3.3. Cd(II) Replacement Reactions with the Zn(II)-HpCdMT Mutants

The so-called “*in vitro* complexes” were obtained by metal displacement reactions using the recombinant Zn-HpCdMT preparations, by adding several molar equivalents of Cd^2+^ ions from a standard solution. As described elsewhere [29], the titrations were performed at pH 7.0, and all assays were performed under an Ar atmosphere. The pH remained constant throughout all experiments without the addition of any extra buffers.

### 3.4. Spectroscopic Analyses (ICP-AES and CD) of the Metal Complexes Formed by the HpCdMT Linker Mutants

The sulfur and metal content of all the metal-MT samples was determined by Inductively Coupled Plasma Atomic Emission Spectroscopy (ICP-AES) in a Polyscan 61E (Thermo Jarrel Ash, Franklin, MA, USA) spectrometer, measuring S at 182.040 nm, Zn at 213.856 nm, and Cu at 324.803 nm. Conventional treatment [30] and incubation in 1 M HNO_3_ at 65 °C for 10 min before measurements to avoid possible traces of labile sulfide anions [31] were used to obtain the protein concentration, by considering that all S atoms were provided by the MT peptides.

CD measurements were performed at 25 °C in a Jasco spectropolarimeter (Model J-715, JASCO, Groß-Umstadt, Germany) interfaced to a computer (J700 software, JASCO, Groß-Umstadt, Germany) by using Peltier PTC-351S equipment (TE Technology, Traverse City, MI, USA). An HP-8453 Diode array UV-VIS spectrophotometer (GIM, Ramsey, MN, USA) was used for the electronic absorption measurements. 1-cm capped quartz cuvettes were employed for spectra recording, and the dilution effects were corrected and processed using the GRAMS 32 software (Thermo Fisher Scientific, Waltham, MA, USA).

### 3.5 Electrospray Ionization Time-of-Flight Mass Spectrometry (ESI-TOF MS) of the Metal Complexes Obtained from the HpCdMT Linker Mutants

A Micro TOF-Q instrument (Bruker Daltonics, Bremen, Germany) interfaced with a Series 1200 HPLC Agilent pump and equipped with an autosampler, all of which controlled by the Compass Software, allowed MW determinations by Electrospray ionization time-of-flight mass spectrometry (ESI-TOF MS). ESI-L Low Concentration Tuning Mix (Agilent Technologies, Santa Clara, CA, USA) was used for calibration. The conditions for the Zn-MT complex analyses were the following: 20 µL of sample solution injected through a PEEK (polyether heteroketone) tubing (1.5 m–0.18 mm i.d.) at 40 µL·min^−1^; capillary counter-electrode voltage 5 kV; desolvation temperature 90–110 °C; dry gas 6 L·min^−1^; spectra collection range 800–2500 *m*/*z*. A 5:95 mixture of acetonitrile:ammonium acetate (15 mM, pH 7.0) was the carrier buffer.

The conditions for the Cu-MT complex analyses were: 20 µL of sample solution injected at 40 µL·min^−1^; capillary counter-electrode voltage 3.5 kV; lens counter-electrode voltage 4 kV; dry temperature 80 °C; dry gas 6 L·min^−1^; and a 10:90 acetonitrile:ammonium acetate 15 mM, pH 7.0 mixture as carrier. The apo-proteins and the Cu-complexes at acid pH were analyzed following the same conditions previously described, but using a liquid carrier consisting of a 5:95 acetonitrile:formic acid mixture at pH 2.4. This causes the release of Zn(II), but keeps Cu(I) bound to the peptides. Experimental mass values were calculated as described in [32], and the error associated with the measurements resulted to be always smaller than 0.1%.

## 4. Conclusions

In summary, in this study we investigated the influence of the polypeptide region linking the two nine-Cys moieties of the CdHpMT isoform, hypothesized to give rise to two independent domains when coordinating divalent metal ions, into the stoichiometry and folding of the corresponding complexes. This was performed by constructing two mutant CdHpMT proteins with longer linkers than the wild-type (eight instead of the two residues), one of them derived from another snail MT sequence: *M. crenulata*, with a clear polar character (HpCdMcMT); and the other derived from a plant MT (the wheat Ec-1 protein), of clear apolar composition (HpCdPlMT). Thereafter, the metal binding behavior of the two mutants was assessed not only for the CdHpMT cognate metal ions (*i.e*., the divalent Zn(II) and Cd(II)), but also for the non-cognate monovalent Cu(I) ions. The results clearly show that both HpCdMcMT and HpCdPlMT form unique Zn_6_- and Cd_6_-complexes, of the same stoichiometry than HpCdMT, and with indistinguishable CD fingerprints. On the contrary, when synthesized in normally-aerated Cu-enriched cultures, the two mutants behave like the wild-type form only in the sense that they yielded a mixture of heterometallic species—expected from their character as non-Cu-thioneins—but otherwise they differed a lot, in comparison to each other as well as in comparison to the wild-type protein. Hence, HpCdMcMT (with a polar linker) yields major M_12_-species (M = Zn or Cu), with Cu_10_ and Cu_11_ cores, and HpCdPlMT (with an apolar linker) yields major M_10_-species (M = Zn or Cu), with a major Cu_8_ core (M = Zn or Cu). Under the same culture conditions, HpCdMT was only capable of folding into major M_5_-species (M = Zn or Cu), with a Cu_4_ core, and only when synthesized at high intracellular Cu concentrations (low culture aeration), similarly to HpCdPlMT major M_10_-species (M = Zn or Cu) with Cu_8_ cores were observed. No Cu(I)-MT complexes were obtained for the mutants at low oxygenation conditions, a fact that is in agreement with the observation that Cu(I)-complexes formed by Cu-thioneins unfold at high Cu concentrations, as has been described for *Drosophila* MtnE [33] or even *M. crenulata* MT [24].

To summarize, variation of the linker (length and amino acid features) does not alter the divalent metal ion (Zn(II) or Cd(II)) binding behavior of HpCdMT, probably because of the independence of the two separate domains. In contrast, variation of the linker (length and sequence) has an influence on the Cu(I) binding behavior of this MT, so that the mutants with the elongated linker better bind Cu(I) ions compared to the wild-type form. Moreover, the more polar the linker, the higher the Cu-thionein character of the protein, as shown by the higher nuclearity and the higher Cu *vs.* Zn content of the heterometallic species resulting from biosynthesis in Cu-enriched cultures. Interestingly, the *C. aspersus* Cu-specific MT (CaCuMT), which has a four-amino acid linker, exhibits a better Cu-binding behavior than the orthologous HpCuMT isoform, which possesses a two-residue linker [34]. This fact is worth considering even if Cu(I) is the non-cognate metal ion for this isoform, because it can provide valuable information about the determinants of the Cu(I) binding capabilities of a great number of MTs with intermediate Zn- *vs.* Cu-thionein character [8].

## Supplementary Materials

Supplementary materials can be found at http://www.mdpi.com/1422-0067/17/01/0006/s1.
