# Supplementary material for: Does Variation of the Inter-Domain Linker Sequence Modulate the Metal Binding Behaviour of Helix pomatia Cd-Metallothionein?
Source: Int J Mol Sci. 2015 Dec 22;17(1):6. doi: 10.3390/ijms17010006 (PMC4730253; doi:10.3390/ijms17010006)
Supplement: Supplementary file 1 [file ijms-17-00006-s001.pdf]

Supplementary Materials: Does Variation of the Inter-Domain Linker Sequence Modulate the Metal Binding Behaviour of *Helix pomatia* Cd-Metallothionein?

Selene Gil-Moreno, Elena Jiménez-Martí, Òscar Palacios, Oliver Zerbe, Reinhard Dallinger, Mercè Capdevila and Sílvia Atrian

---

HpCdMT: wild type *Helix pomatia* CdMT

HpCdMT cDNA sequence (with short linker highlighted in blue): 220pb

TTTATTGGATCCGGAAAAGGAAAAGGAGAAAAGTGCACCTCAGCTTGCAGGAGCGAGCCTTGC
CAGTGTGGGAGCAAGTGCCAGTGTGGGGAAGGCTGTACATGTGCCGCCTGCAAGACTTGCAAC
TGTACCAGTGATGGTTGCAAGTGCGGCAAGGAGTGCACCGGCCCAGACTCGTGCAAGTGTGGC
AGCTCGTGCAGCTGCAAGTAACTCGAGAAAA

HpCdMT protein sequence:

MSGKGKGEKCTSACRSEPCQCGSKCQCGEGCTCAACKTCNCTSDGCKCGKECTGPDS
CKCGSSCSCK*

HpCdMcMT: *Helix pomatia* CdMT with the linker of the *M. crenulata* MT

HpCdMcMT cDNA sequence (linker from *Megathura crenulata* highlighted in pink): 237 pb

TTTATTGGATCCGGAAAAGGAAAAGGAGAAAAGTGCACCTCAGCTTGCAGGAGCGAGCCTTGC
CAGTGTGGGAGCAAGTGCCAGTGTGGGGAAGGCTGTACATGTGCCGCCTGCGTGAAGACAGAA
GCCAAAACCACGTGCAACTGTACCAGTGATGGTTGCAAGTGCGGCAAGGAGTGCACCGGCCCA
GACTCGTGCAAGTGTGGCAGCTCGTGCAGCTGCAAGTAACTCGAGAAA

HpCdMcMT protein sequence:

MSGKGKGEKCTSACRSEPCQCGSKCQCGEGCTCAACVKTEAKTTCNCTSDGCKCGKE
CTGPDSCKCGSSCSCK*

HpCdPlMT: *Helix pomatia* CdMT with the linker of the *Ec-1* wheat MT

HpCdPlMT cDNA sequence (linker from the *Ec-1* wheat highlighted in green): 237 pb

TTTATTGGATCCGGAAAAGGAAAAGGAGAAAAGTGCACCTCAGCTTGCAGGAGCGAGCCTTGC
CAGTGTGGGAGCAAGTGCCAGTGTGGGGAAGGCTGTACATGTGCCGCCTGCTCGGCGAGGAGC
GGCGCGGCGGCGTGCAACTGTACCAGTGATGGTTGCAAGTGCGGCAAGGAGTGCACCGGCCCA
GACTCGTGCAAGTGTGGCAGCTCGTGCAGCTGCAAGTAACTCGAGAAA

HpCdPlMT protein sequence:

MSGKGKGEKCTSACRSEPCQCGSKCQCGEGCTCAACSARSGAAACNCTSDGCKCGK
ECTGPDSCKCGSSCSCK*

Figure S1. cDNA and protein sequence information of the proteins studied in this work. The constructs HpCdMcMT and HpCdPlMT, in comparison with the HpCdMT wild type form. The cDNA and protein sequence corresponding to the linker segments are colored, and the grey boxes indicate the 5′ and 3′ restriction sites (*BamH*I and *Xho*I, respectively) used for cloning into the pGEX-4T-1 plasmid.
